# Annotations

**Published:** 1902-02-15

**Authors:** 


### Annotations.

### The Break-up of the Victoria University.

The multiplication of universities is by no means an unmixed benefit, and we foresee very definite danger in the proposals which are now taking a concrete form for tearing in pieces that somewhat artificial product, the Victoria University, and establishing three separate universities in its stead. So far as teaching is concerned, the more the merrier. Once get rid of the false idea of university functions, which for the last half-century has been imposed upon us by the University of London, namely, that examinations are the beginning and end of all things, and we revert to the older conception of a university as a seat of learning, a resort of scholars, a centre for that best form of education which consists in association with leaders of thought and pioneers of investigation in the daily occupations of their lives. Such universities must always be local; their advantages lie in their teaching rather than in their degrees; the benefits they confer fall not only upon their students and professors alone, but upon the cities in which they have their homes. Of such universities we cannot have too many. But a university which is a degree shop is another matter, and no one can think of the increasing number of institutions which will soon be engaged in distributing degrees, and, what is worse, in making their living by the process, without wondering what will ultimately be the value of the various letters which we shall soon see appended to everybody's name. As concerns arts, divinity, and music, a certain comic element enters into this wide scattering of distinctions, which takes away from the gravity of the situation. Medicine, however, stands on a different footing, and so far as the conferring of medical degrees confers also the right of admission to the Register, and thus the right to practice on their fellow-countrymen, we cannot but regard with considerable anxiety the proposed multiplication of portals to the medical profession. That each medical school should be so connected with a university as to offer a medical degree to such of its alumni as go creditably through its course, is sufficiently obvious; but that the ways of access to the Register should be multiplied is by no means so clear. The end must come sooner or later, and such a large multiplication of universities as is now suggested will probably drive us a long way towards admitting the necessity of a single State examination as the one sole portal to the practice of medicine.

### The London Water Supply.

The Government proposals for the creation of a water authority which shall not act for, or be representative of Central London alone, but shall include the whole of what is termed Water London, running far out into the surrounding counties, as indeed the operations of water companies themselves do, has raised many protests from the more "advanced" school of London politicians, who maintain that the London County Council as the appointed representatives of the people ought to have the direct control of the water supply. And there would be much to say for such an argument did the County Council in fact represent the whole of London. No one can doubt that the provision of pure water is one of the first and most important factors in maintaining health in any community, and that the control of all the arrangements for maintaining a high standard, both in regard to the quantity and purity of the supply, ought to be one of the first and most important duties of its representatives. On the other hand, no one who has watched the growth and especially the outward growth of London can fail to see that just as the City is but a patch in the centre of the County, so the County is rapidly becoming but a patch in the centre of a large and highly populated district which, although outside the boundaries and independent of the control of London, really constitutes the London in which Londoners live. What is evident is that the existing boundaries of the Administrative County of London are but a makeshift, and that the logical sequence of the creation of the County ought to be its wide extension. For a long time back London has been bursting its bounds on every side, and there is every probability that with the introduction of new modes of locomotion it will soon spread itself out in a manner hitherto undreamed of, and in arranging for the water supply what is important is not merely to find the best and most representative authority as things stand at present, but so to arrange matters as not to create new vested interests, and not to set up new obstacles in the way of that extension of the boundaries of the County, and that enlargement of the powers of the Council which will shortly become necessary for the proper sanitary administration of the metropolis.

### The Limits of "Covering."

The *British Medical Journal* is doing good service in exposing the nature of certain "cures" for alcoholism which have lately come into vogue, "cures" which, judging by the fees charged and the number of cases which are said to be treated, must be veritable gold mines to their proprietors. We wish the journal every success in its mission, although we do not believe that it will do a ha'porth of good. The sort of people who love quackery will get quacked somehow or other, whatever sensible people may say. There is, however, a side of the question to which, we think, attention should be drawn, and that is, the public statement that certain of these "cures," which involve treatment by drugs, although carried out by persons outside the pale of the profession, are performed under the supervision and the sanction of qualified medical practitioners. The General Medical Council has done much to force practitioners to walk strictly in the path of professional rectitude. It has not hesitated to turn adrift the unqualified assistant, or to deprive practitioners of their livelihood for the professional crime of associating themselves with unqualified persons in carrying on medical practices. Of late it has gone so far as to declare it "infamous in a professional respect" for a doctor to associate himself in practice with any dentist who is not on the register, even by giving chloroform for him while he pulls a tooth! What, then, is to be done with medical men who associate themselves with "cures" for the "treatment" of alcoholism conducted by non-medical and, of course, unregistered people? For the matter of that, what is to be done with doctors who associate themselves with any sort of medical "treatment" carried out by unqualified people? Does such association amount to "covering," in other words, does it give such a savour of medical authority as to delude the public?

